# The Role of AOPP in Age-Related Bone Loss and the Potential Benefits of Berry Anthocyanins

**DOI:** 10.3390/nu9070789

**Published:** 2017-07-22

**Authors:** Melissa M. Melough, Xin Sun, Ock K. Chun

**Affiliations:** Department of Nutritional Sciences, University of Connecticut, Storrs, CT 06269, USA; melissa.melough@uconn.edu (M.M.M.); xin.sun@uconn.edu (X.S.)

**Keywords:** AOPP, bone, aging, osteoporosis, antioxidants, berry anthocyanins

## Abstract

Age-related bone loss is a major factor in osteoporosis and osteoporotic fractures among the elderly. Because bone homeostasis involves a balance between bone formation and resorption, multiple mechanisms may induce age-dependent changes in bone. Oxidative stress is one such factor that contributes to the pathology of aging-associated osteoporosis (AAO). Advanced oxidation protein products (AOPP) are a biomarker of oxidant-mediated protein damage, and can also act to increase the production of reactive oxygen species (ROS), thereby perpetuating oxidative damage. AOPP is a relatively novel marker of oxidative stress, and its role in bone aging has not been fully elucidated. Furthermore, it has been theorized that dietary antioxidants may decrease AOPP levels, thereby reducing AAO risk, but a limited number of studies have been specifically targeted at addressing this hypothesis. Therefore, the objective of this review is to examine the findings of existing research on the role of AOPP in age-related bone loss, and the potential use of dietary antioxidants to mitigate the effects of AAOP on age-related bone loss. Cross-sectional studies have delivered mixed results, showing that AOPP levels are inconsistently associated with bone loss and aging. However, in vitro studies have documented multiple mechanisms by which AOPP may lead to bone loss, including upregulation of the JNK/p38 MAPK signaling pathways as well as increasing expression of sclerostin and of receptor activator of NFκB ligand (RANKL). Studies also indicate that antioxidants—especially berry anthocyanins—may be an effective dietary agent to prevent aging-associated bone deterioration by inhibiting the formation of AOPP and ROS. However, the understanding of these pathways in AAO has largely been based on in vitro studies, and should be examined in further animal and human studies in order to inform recommendations regarding dietary anthocyanin use for the prevention of AAO.

## 1. Introduction

Age-related bone loss is a primary contributor to osteoporosis and osteoporotic fractures in the elderly. Osteoporosis is considered a major public health threat for an estimated 44 million Americans, or 55% of the population aged 50 years and older [2]. Numerous studies employing various methods have dealt with the pathophysiology of postmenopausal osteoporosis [3], but aging-associated osteoporosis (AAO) has not been as well studied [4]. AAO is thought to be a type of low-turnover osteoporosis resulting from aging-associated calcium deficiency and an imbalance between bone resorption and formation [5].

Oxidative stress plays a central role in human aging and accelerates the aging process [6,7]. Bone density is maintained by two phases of bone remodeling: bone resorption by osteoclasts and bone formation by osteoblasts [8]. Emerging evidence indicates that the increased production of reactive oxygen species (ROS) in bone cells may activate bone resorption, resulting in a gradual decline in bone mass and density with aging [7]. Studies have shown that oxidative stress results in reduced bone formation, increased osteoblast and osteocyte apoptosis, and decreased bone mineral density (BMD) in aged mice [9]. Reduced BMD is one of the defining characteristics of osteoporosis, and correlates with bone strength and fracture risk [2]. Induced oxidative stress in young mice and rats has also been shown to reduce osteoblastogenesis and to increase osteoclast number and activity [10,11]. Loss of sex steroids potentiates the effects of aging by weakening defense mechanisms against oxidative stress [10]. Previous studies have clearly demonstrated that the bone turnover pattern remains relatively steady in advanced aging [12]. Based on this pattern, there is a reduction in bone formation as a result of a decreased recruitment of osteoblasts and an elevation of bone resorption that might result from enhanced activity of osteoclasts, which is the primary underlying mechanism of AAO [12].

**Figure 1:**
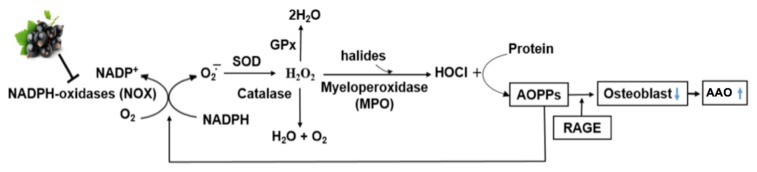
Potential mechanism of anthocyanins in lowering aging-associated osteoporosis (AAO) risk through inhibiting nicotinamide adenine dinucleotide phosphate (NADPH) oxidase (NOX)-mediated advanced oxidation protein products (AOPP) formation. GPx: glutathione peroxidase; RAGE: receptor for advanced glycation end products; SOD: superoxide dismutase.

Advanced oxidation protein products (AOPP) arise from the reaction between plasma proteins and chlorinated oxidants (e.g., hypochlorous acid, HOCl) by the H_2_O_2_-myeloperoxidase (MPO) system, and are di-tyrosine-containing cross-linking protein products considered to be novel markers of oxidant-mediated protein damage [13,14,15]. AOPP are mainly carried by albumin in the circulation, and oxidized albumin is rapidly cleared mainly through the uptake by the liver and spleen [16]. Like advanced glycation end products (AGEs), AOPP signal via the receptor for AGE (RAGE) in endothelial cells and induce endothelial dysfunction [17]. Increased plasma levels of AOPP have been found in many diseases, such as diabetes, uremia, obesity, coronary artery disease, and inflammatory bowel diseases. Evidence indicates that AOPP increase with age, and that AOPP can trigger cytosolic superoxide generation via the activation of nicotinamide adenine dinucleotide phosphate (NADPH) oxidase (NOX), which is a major source of ROS [18] (Figure 1). Therefore, AOPP may serve not only as markers of oxidant-mediated protein damage, but also as potential inducers of oxidative stress [15].

Due to the public health relevance of AAO and the emerging evidence demonstrating the role of oxidative stress in bone aging, it is of great importance to characterize the ways in which AOPP may affect bone health and homeostasis. It has also been hypothesized that due to their antioxidant capacity, anthocyanins found in plants such as berries may prevent or reduce bone resorption and deterioration. Therefore, the purpose of this review is to examine the current state of knowledge regarding the role of AOPP in age-related bone loss and to assess the potential for the use of berry anthocyanins to reduce the formation of AOPP and improve bone-related outcomes in aging.

Articles included in this review were identified using PubMed and Web of Science. To examine the role of AOPP in bone loss, databases were searched for “AOPP bone loss”. Other keywords added to this search were osteoporosis, osteoblasts, and osteoclasts. To find studies investigating the effects of anthocyanins on bone loss, databases were searched for “berry anthocyanin bone loss”, and the following terms were added to expand the search: AOPP, antioxidant, osteoporosis. All articles for this review were published by May 2017. Publications identified by these methods were then limited to those describing cross-sectional analyses of AOPP and bone health in humans and animals, in vitro studies of cells treated with AOPP, and clinical trials or observational studies seeking to examine the effects of dietary antioxidants on AOPP and bone outcomes.

## 2. Aging-Associated Osteoporosis

Osteoporosis is a skeletal disease characterized by the deterioration of bone mass and microarchitecture, leading to increased fragility and predisposition to bone fractures [2]. While postmenopausal osteoporosis is related to estrogen deficiency and affects trabecular bone, aging-associated osteoporosis (AAO) primarily affects cortical bone and has many contributing factors including genetics, nutrition, physical activity, and physiological changes to bone [5]. Because bone loss appears to begin near age 40 and accelerates after age 60, age-related bone loss affects a growing number of men and women as the global population of older individuals increases [19].

One well-established physiological effect of bone aging is decreased bone mineral content, which is associated with increased brittleness and decreased fracture resistance [20]. Aging is also associated with morphological changes to bone such as thinning of the cortical walls and the overall slimming of the bones, as well as changes to proteins in bone, including collagen [20]. Increased bone resorption in aging may also contribute to reduced bone mineral density (BMD), and can be exacerbated by reductions in bone formation related to reduced osteoblastogenesis and increased adipogenesis in the bone marrow, which affects matrix formation and mineralization [21]. The etiology of osteoporosis is multifactorial and influenced by both genetic and environmental factors. Of particular interest to this review is the effect of oxidative stress.

## 3. AOPP as a Marker of Oxidative Stress in Bone

Studies indicate that oxidative stress may enhance bone resorption and disturb the coupling of bone resorption to bone formation, contributing to AAO [19,22]. Oxidative stress increases with age, as ROS production rises and the activities of antioxidant enzymes such as superoxide dismutase (SOD) and glutathione peroxidase simultaneously decrease [19,23]. The oxidative stress resulting from this imbalance can stimulate apoptosis of osteoblasts and osteocytes [24,25], and may reduce osteoblastogenesis [26] while also increasing the formation and activation of osteoclasts [10].

AOPP is a novel marker of oxidative stress that may be particularly important in the context of bones, both as a biological driver and a biomarker of bone degradation. Several cross-sectional studies have examined the relationship between bone status and AOPP in both humans and animals (Table 1). Zhang et al. demonstrated that among male Wistar rats, AOPP levels in both plasma and femurs increased with age, while SOD activity decreased [19]. AOPP was also associated with decreases in BMD, bone volume, trabecular thickness, and the rate of bone formation. Similarly, a 2015 study showed that plasma AOPP was associated with reduced BMD and increased markers of bone turnover among postmenopausal women [27]. Importantly, this study did not adjust for potential cofounding factors such as age, body mass index, diet, or smoking. Recent studies by a group in Italy linked lipid hydroperoxides—one marker of oxidative stress—to reduced BMD in postmenopausal women, but did not find significant relationships between AOPP and BMD [28]. Differences between these studies could reflect differences in study populations as well as the progression of bone disease or oxidative damage. The currently available cross-sectional study data provide weak evidence to support the link between AOPP and bone health. Given these mixed findings and important limitations to each study, alternative study designs that illuminate potential mechanisms of AOPP in bone are essential for understanding the relationship between AOPP and osteoporosis.

Few interventional trials in animals have investigated the actions of AOPP on bone. One study showed that AOPP administration accelerated bone deterioration in aged male rats and that the AOPP-induced changes in bone turnover markers, trabecular BMD, and microstructural parameters could be completely prevented by the oral administration of the NOX inhibitor apocynin, suggesting that AOPP induced bone deterioration via the activation of NAPDH oxidase [4]. Another study showed that administration of the radical scavenger antioxidant melatonin reduced AOPP levels and other markers of oxidative stress in streptozotocin-induced diabetic male rats, leading to beneficial effects on bone healing in a short-term study [29]. Although not specific to osteoporosis, this suggests that AOPP may inhibit bone formation.

## 4. Mechanisms of Action of AOPP in Bone

Several in vitro studies that have challenged cells with AOPP have helped to clarify the mechanisms by which AOPP may impact bone (Table 2). A recent study using an osteocyte-like cell line (MLO-Y4) demonstrated that when cultured with AOPP-modified mouse serum albumin (AOPP-MSA), AOPP increased ROS generation and activated the c-Jun N-terminal kinase (JNK) and p38 mitogen-activated protein kinase (p38 MAPK) signaling pathways in a ROS-dependent manner, leading to apoptosis of these cells [30]. Upregulation of the JNK/p38 MAPK signaling pathways by AOPP also increased the expression of the protein sclerostin [30], which inhibits osteoblast function and bone formation by antagonizing the Wnt signaling pathway [31]. Through the JNK/p38 MAPK pathway, AOPP also upregulated the expression of receptor activator of NFκB ligand (RANKL) [30], which can upregulate osteoclastogenesis upon binding to its receptor [32].

A study using rat mesenchymal stem cells (MSC, which give rise to osteoblasts and osteocytes) demonstrated that exposure to AOPP-modified bovine serum albumin (AOPP-BSA) inhibited MSC proliferation, reduced alkaline phosphatase (ALP) activity, decreased collagen I mRNA levels, and inhibited bone nodule formation [33]. AOPP also increased ROS generation and upregulated the expression of RAGE [33]. In rat osteoblast-like cells, AOPP-modified rat serum albumin (AOPP-RSA) induced many of the same effects and provided evidence that AOPP may inhibit the proliferation of osteoblast-like cells through the ROS-dependent NFκB pathway [34].

Taken together, these in vitro studies indicate that AOPP may act through the NFκB, JNK, and p38 MAPK signaling pathways to inhibit bone formation while promoting resorption. Previous reports have shown that the binding of RANKL to RANK causes recruitment of TNF receptor-associated factor 6 (TRAF6), which in turn activates NFκB, JNK, and MAPK, which induce nuclear factor of activated T cells (NFAT)c1, a key transcription factor for osteoclastogenesis [35,36]. Therefore, AOPP may disturb the balance between bone formation and resorption by upregulating osteoclastogenesis through these pathways.

Furthermore, it has also been documented that AGE-RAGE interaction induces the generation of ROS through the NOX pathway, resulting in the apoptosis of osteoblasts/MSC [37] and in the inhibition of the proliferation and differentiation of osteoblasts/MSC [38]. RAGE overexpression by lentiviral transfection has been shown to inhibit osteoblast proliferation through the suppression of the Wnt, phosphoinositide 3-kinase (PI3K), and extracellular signal-related kinase (ERK) pathways [38]. It has also been reported that RAGE plays a critical role in osteoclast maturation and activation [39], and RAGE expression in osteoclasts is age-dependent [40]. Studies using RAGE knockout mice have also shown that bone mass and bone biomechanical strength are increased with a decreased number of osteoclasts compared with wild-type mice [41]. Therefore, based on observations from the current literature, AOPP may inhibit osteoblastic activity and differentiation through the AOPP-RAGE-ROS pathway via the activation of NOX, which may be an important mechanism involved in the development of AAO.

## 5. Antioxidant Intake and Bone Health: Potential Benefits of Berry Anthocyanins

Because of the potential role of AOPP in aging-associated bone turnover, it is plausible that increased consumption of dietary antioxidants could reduce the formation of AOPP by inhibiting the NOX pathway, thereby lowering AAO risk. Two studies that support this hypothesis showed that murine osteoblastic MC3T3-E1 cells cultured with 2-deoxy-d-ribose (dRib) to induce oxidative damage could be rescued from dRib toxicity by the addition of the flavonoid antioxidants hesperetin [41] and myricetin [42], and that these antioxidant treatments markedly reduced AOPP along with other markers of oxidation. Melatonin is a well-studied antioxidant [43], and has also been shown to protect against H_2_O_2_-induced apoptosis of MSC [44]. The positive effects of dietary antioxidants on BMD and bone status have also been demonstrated in multiple cross-sectional investigations [45,46,47]. However, due to the inherent limitations of cross-sectional studies, this hypothesis should be further investigated using cohort studies or interventional trials.

Recently, considerable attention has been directed to the potential favorable effects of berries in enhancing bone health due to the antioxidant properties of anthocyanins in berries. Several studies indicate that blackcurrant anthocyanins exhibit a range of health benefits, including antioxidant [48,49] and anti-inflammatory effects [50,51], which could potentially improve bone remodeling. Several studies have utilized ovariectomized (OVX) animals to mimic the estrogen deficiency of menopause, and have found that supplementation with blueberry or blackcurrant attenuated the OVX-induced bone loss (Table 3) [52,53,54].

However, evidence of bone-protective effects of berries outside of an estrogen-deficient model is still limited. In recent in vitro experiments using murine bone marrow macrophages, anthocyanins from blackcurrant, blackberry, and blueberry suppressed NOX (NOX1 and NOX2) mRNA expression by over 60% [51]. This reduction consequently downregulated nuclear factor (erythroid-derived 2)-like 2 (Nrf2) mRNA expression, suggesting that the NOX pathway was the major source of ROS production and that berry anthocyanins effectively inhibited the NOX pathway, thus reducing ROS production (Figure 1). In cultured RAW 264.7 macrophages, anthocyanins from blackcurrant, blueberry, and blackberry significantly inhibited lipopolysaccharide-induced inflammation as indicated by lower mRNA levels of TNFα and interleukin-1β, and lowered nuclear p65 levels, indicating decreased NFκB activity [51]. TNFα plays a central role in inflammation-mediated bone loss by augmenting osteoblastic RANKL-induced osteoclastogenesis and directly stimulates osteoclast formation [55,56]. These results indicate that berry anthocyanins may be an effective dietary agent in preventing aging-associated bone deterioration directly by inhibiting NOX-mediated AOPP formation and indirectly by reducing bone resorption through lowering ROS formation.

## 6. Conclusions

Oxidative stress contributes to the universal phenomenon of bone aging, and is a key factor in the development of AAO. AOPP is a biomarker of oxidative damage to protein and has been associated with lower BMD in both humans and animals in some observational studies. While not all observational studies confirmed the role of AOPP in AAO, the association between AOPP and bone loss is supported by several mechanistic studies elucidating the signaling pathways by which AOPP may reduce bone formation and/or increase bone resorption. Relatively little work has specifically examined how dietary antioxidants may impede bone aging through the reduction of AOPP. Studies that have addressed this hypothesis indicate that antioxidant consumption may be an effective method of inhibiting AOPP formation and lowering ROS formation in bone. Importantly, these findings are largely based on in vitro studies and should be expanded in future research examining how long-term consumption of dietary antioxidants reduces AOPP formation and mitigates aging-associated bone loss in older adulthood. This type of research may serve as a basis for future human clinical studies, which may ultimately lead to the development of dietary recommendations and strategies for the prevention of AAO.

## Figures and Tables

**Table 1:** Cross-sectional studies of AOPP and bone status in humans and animals.

Study	Population	Observations	Limitations
Zhang (2011) [19]	Young, adult, and old (*n* = 26 each) male Wistar rats	AOPP in plasma and femur increased with aging and were negatively associated with femur BMD	Sample size; potentially limited translatability to humans
Wu (2015) [27]	60 postmenopausal women with osteoporosis, 60 without osteoporosis	AOPP was associated with reduced BMD and increased bone turnover markers	Sample size; no adjustment for factors such as BMI, diet, or smoking; BMD assessed only at lumbar spine
Cervellati (2013) [28]	98 pre- and 93 post-menopausal women	No significant association between AOPP and bone status	Potential for residual confounding; AOPP assessed only in serum
Cervellati (2014) [22]	167 postmenopausal women	No significant association between AOPP and bone status	Potential for residual confounding; AOPP assessed only in serum

AOPP: advanced oxidation protein products; BMD: bone mineral density; BMI: body mass index.

**Table 2:** In vitro studies of the effect of AOPP on bone cells.

Study	Cell Type	Treatments	Outcome
Yu (2016) [30]	Osteocytic MLO-Y4 cells	Cultured with AOPP-MSA (25, 50, 100, or 200 μg/mL for 24 h or 200 μg/mL for 3, 6, 12, or 24 h)	AOPP triggered apoptosis and upregulated expression of sclerostin and RANKL in a JNK/p38 MAPK-dependent manner
Sun (2013) [33]	Rat MSC	Cultured with AOPP-BSA (50, 100, 200, or 400 μg/mL for 3 days or 200 μg/mL for 24, 48, or 72 h)	AOPP inhibited proliferation, reduced ALP activity and ALP and collagen I mRNA, increased ROS generation, upregulated RAGE expression
Zhong (2009) [34]	Rat osteoblast-like cells	Cultured with AOPP-RSA (50, 100, or 200 μg/mL for 24 h or 100 μg/mL for 24, 48, or 72 h)	AOPP inhibited proliferation, reduced ALP activity, downregulated expression of osteocalcin, induced ROS generation and NFκB phosphorylation

AOPP: advanced oxidation protein products; MLO-Y4: murine osteocyte-like cell line Y4; AOPP-MSA: AOPP-modified mouse serum albumin; RANKL: receptor activator of NFκB (nuclear factor κB) ligand; JNK: c-Jun N-terminal kinase; p38 MAPK: p38 mitogen-activated protein kinase; MSC: mesenchymal stem cells; AOPP-BSA: AOPP-modified bovine serum albumin; ALP: alkaline phosphatase; ROS: reactive oxygen species; RAGE: receptor for advanced glycation end products; AOPP-RSA: AOPP-modified rat serum albumin.

**Table 3:** Animal studies of the impact of berry antioxidants on ovariectomy-induced bone loss.

Study	Population	Treatments	Duration	Outcome
Li (2014) [52]	Female Sprague Dawley rats (total *n* = 30)	Randomized to sham operation, OVX control, and OVX blueberry treatment (10% *w*/*w* freeze-dried blueberry powder)	12 weeks	Blueberry inhibited bone resorption, bone loss, and the reduction of bone strength of OVX rats
Zheng (2016) [53]	Female C57BL/6J mice (total *n* = 54)	Randomized to sham operation or OVX, then further divided into control diet or diet containing 1% blackcurrant extract	4, 8, or 12 weeks	Blackcurrant attenuated OVX-induced bone loss as measured by BMD and trabecular volume; blackcurrant reduced bone resorption activity
Devareddy (2008) [54]	Female Sprague Dawley rats (*n* = 30)	Randomized to sham operation, OVX control, and OVX blueberry treatment (5% *w*/*w* dried blueberry powder)	100 days	Blueberry prevented OVX-induced loss of whole-body BMD; blueberry treatment group had lower serum osteocalcin

OVX: ovariectomized; BMD: bone mineral density.
